# Effects of Different Training Methods on Open-Skill and Closed-Skill Agility in Basketball Players: A Systematic Review and Meta-Analysis

**DOI:** 10.1186/s40798-025-00842-9

**Published:** 2025-05-07

**Authors:** Mingxiang Zhang, Feng Li, Jiao Jiao, Wei Liang, Miguel-Angel Gomez, Aaron T. Scanlan

**Affiliations:** 1 China Basketball College, Beijing Sport University, Beijing, China; 2 Department of Sport, Physical Education and Health, Hong Kong Baptist University, Hong Kong, China; 3 School of Physical Education, Shenzhen University, Shenzhen, China; 4 Faculty of Physical Activities and Sport Sciences, Universidad Politécnica de Madrid, Madrid, Spain; 5 School of Health, Medical and Applied Sciences, Central Queensland University, Rockhampton, QLD Australia

**Keywords:** Change-of-direction, Reaction Training, Plyometric, Speed, Strength, Balance, Stretching

## Abstract

**Background:**

Open-skill and closed-skill agility attributes are pivotal for achieving success in basketball. However, systematic synthesis of evidence regarding the effectiveness of different basketball-specific training methods on agility performance is lacking among basketball players in the literature. Consequently, this systematic review with meta-analysis aimed to evaluate the effectiveness of prominent training methods in improving open-skill and closed-skill agility in basketball players.

**Methods:**

Using keywords related to ‘basketball’, ‘agility’, and ‘training’, we searched for experimental studies in PubMed, Web of Science, and EBSCOhost databases that were published in the last decade (between January 2013 and September 2023). The included training methods were categorized into five groups, including reaction training (RT), speed training (SpT), strength and balance training (SBT), plyometric training (PT), and stretching training (StrT). The effects of training methods were summarized using standardized mean differences (SMD) with 95% confidence intervals in R software.

**Results:**

A total of 29 studies met the inclusion criteria, comprising 42 separate effects. Studies only assessed the effects of different training methods on closed-skill agility performance, with no open-skill agility assessments used. Improvements in closed-skill agility were apparent between pre-and post-training intervention with most training methods including a large effect for RT [SMD = 0.86, 95% CI (0.53, 1.19)], medium effects for PT [SMD = 0.62, 95%CI (0.38, 0.86)] and SBT [SMD = 0.59, 95%CI (0.13, 1.05)], and a small effect for SpT [SMD = 0.43, 95%CI (0.13, 0.74)]. While no effect for StrT [SMD = 0, 95%CI (-0.98, 0.98)] was apparent, only one study examined this training method.

**Conclusions:**

RT appears to be the most effective method for developing closed-skill agility among basketball players, particularly when implemented in small-sided games. SBT and PT also appear impactful in developing closed-skill agility to similar extents. SpT appears to benefit closed-skill agility to a minor extent with limited research examining the effectiveness of StrT on agility among basketball players. Surprisingly, no studies have incorporated open-skill agility tests when assessing the effectiveness of training methods, which is essential to address in future research. Outcomes from this review provide guidance to basketball coaches and performance staff for selecting training methods that optimize closed-skill agility performance in their players.

## Key Points

Reaction training appears to be the most effective method in enhancing closed-skill agility performance among basketball players, predominantly administered in the form of small-sided games.

Plyometric, strength, and balance training may also be effective components to include in training programs when seeking to optimize closed-skill agility performance in basketball players.

Training methods focused solely on speed development offer limited benefit to closed-skill agility performance in basketball players.

The effects of different training methods on open-skill agility performance remain largely unknown given the lack of studies assessing this attribute, highlighting the need for future research attention in this area.

## Background

Agility is a critical fitness attribute in basketball given it is heavily utilized in executing various offensive and defensive tasks [1]. In this regard, players need to frequently execute change-of-direction (COD) movements in confined spaces, which can be pre-planned in nature or in reaction to stimuli. Consequently, agility performance is thought to depend on perceptual and decision-making factors (i.e., visual scanning, anticipation, pattern recognition, and knowledge of situations) as well as COD speed (which depends on technical ability, linear speed, as well as strength, power, and reactive strength qualities) [2]. Considering these cognitive and physical constituents, agility performance has been sub-categorized into open-skill and closed-skill agility [3–5]. More precisely, open-skill agility involves movement being performed across an unplanned path in response to relevant stimuli, encompassing both cognitive and physical attributes [4]. In contrast, closed-skill agility involves movement being performed across a pre-planned path, focusing more on physical attributes in changing direction and/or velocity [4, 5]. Despite this distinction, agility has typically been defined primarily in terms of its physical components, with less emphasis on its cognitive demands [3]. In the present review, we conceptualize agility as a multidimensional ability that integrates both cognitive and physical components. This perspective is crucial for distinguishing between the training effects on open-skill and closed-skill agility.

In basketball, male players execute over 1,000 actions per game, with a new action being performed every 2 s on average and often being multidirectional in nature [6, 7]. In this way, players may need to rapidly change direction and/or velocity to outmaneuver opponents and create scoring opportunities on offence as well as to remain in position to guard opponents or secure loose balls on defence [1, 8–10]. Consequently, basketball coaches and performance staff place strong emphasis on incorporating agility-oriented training methods within the annual plan to optimize player performance [11]. Moreover, agility training may offer wider benefits beyond performance in reducing injury risk among players by enhancing body control, equilibrium, and coordination, thereby helping to protect player health and availability to compete [12].

Despite the inherent importance for delivering agility training among basketball practitioners, alongside the growing body of research exploring the effectiveness of different agility training methods in basketball players, only one systematic review has been conducted on this topic to date in basketball [13]. Specifically, Wang et al. [13] examined the impact of various training methods on the agility of basketball players, analyzing 26 studies with 553 participants. The authors found that reaction training methods yielded the greatest improvements in agility, with plyometric training also enhancing agility, while speed and strength training had minimal effects on agility performance [13]. However, despite providing novel insight into the effectiveness of different training methods on agility in basketball players, there were some notable limitations within this previous review. Firstly, the authors did not distinguish between open-skill and closed-skill agility when defining agility nor when referring to the type of performance assessed surrounding training interventions in studies. Given open-skill and closed-skill agility have been shown to be independent attributes [14, 15], it is essential that the effects of different training methods on each type of agility are delineated. Secondly, limited practical insight can be inferred from this previous review due to the lack of descriptions provided for the different training programs adopted within studies. Thirdly, the precise effects that each training method may exert on agility performance cannot be elucidated in this previous review given the authors did not conduct analyses to quantify the magnitudes of training effects across studies. Consequently, understanding of the most effective training methods for enhancing open-skill and closed-skill agility in basketball remains to be definitively reported across the existing literature. Accordingly, this systematic review and meta-analysis aimed to quantify the effectiveness of the predominant methods adopted in research to train agility performance in basketball players. While the primary focus of this review is on the overall agility performance outcomes, the meta-analysis also incorporates studies assessing agility in both ball-handling and non-ball-handling contexts where applicable. The findings of this review will guide training strategies adopted by basketball coaches and performance staff in practice to optimize agility performance among their players.

## Methods

### Literature Retrieval Strategy

A systematic review and meta-analysis was conducted following the guidelines outlined in the Preferred Reporting Items for Systematic Reviews and Meta-Analyses (PRISMA) 2020 statement [16]. The review also adhered to the PROSPERO guidelines and was registered at PROSPERO (CRD42023391822). Two reviewers developed the search strategies and searched three electronic databases (Web of Science, PubMed, and EBSCOhost) to identify original research studies focusing on agility training in basketball. The following search string was adopted in PubMed and EBSCOhost: (basketball) AND (agility OR dexterity OR lower limb flexibility OR agile OR nimble) AND (training OR drill). The following search string was applied in Web of Science: (TS=(basketball)) AND (TS=(agility OR dexterity OR lower limb flexibility OR sensitiveness OR sensitive OR agile OR nimble)) AND (TS=(training OR drill)). Retrieved studies were restricted to being published between January 2013 and September 2023 to include the most recent developments and insights in the field from the past decade.

### Literature Inclusion and Exclusion Criteria

The inclusion criteria for studies in this review were determined using the PICOS framework [17] as follows: (1) Population: adolescent and adult basketball players aged 12 years and older [18]; (2) Intervention: any exercise-based training method (including physical or cognitive interventions) lasting at least 4 weeks; (3) Comparison: within-group comparisons; (4) Outcomes: assessment of open-skill or closed-skill agility performance before and after the training intervention; (5) Study design: experimental or quasi-experimental trials, including randomized controlled trials or crossover trials. The following exclusion criteria were applied: (1) studies published in languages other than English; (2) studies for which full-text versions could not be retrieved; (3) studies recruiting amateur basketball players (Tier 0 or Tier 1 according to a participant classification framework) [19]; (4) studies that do not clearly describe the intervention or outcome measures; (5) studies that include additional interventions (e.g., nutritional supplements) alongside the primary training program; (6) studies that did not evaluate (or provide data for) agility performance prior to and following the intervention; and (7) studies categorized as low quality based on the quality assessment procedures.

### Quality Assessment and Sensitivity Analysis

Although Delphi, PEDro, and Cochrane are commonly used for quality evaluations, the absence of control groups in many of this review’s studies (13 out of 29) hinders objective assessments of crucial aspects like randomization, participant blinding, and intention-to-treat analysis. Consequently, we chose the Brughelli modified quality assessment form, which is specifically designed to objectively evaluate the quality of training intervention studies [20]. This tool encompasses ten critical factors, each assigned a score ranging from 0 to 2, culminating in a maximum score of 20; where 0 denotes ‘clearly no,’ 1 denotes ‘possibly yes,’ and 2 denotes ‘clearly yes’ for each factor. Two researchers evaluated included studies with a third researcher consulted for a consensus decision where any disagreements could not be resolved via discussion. Based on the outcomes of the quality assessment, studies scoring < 13 were categorized as low quality (and excluded from the review), those scoring from 13 to 16 were deemed to be of medium quality, and those scoring from 17 to 20 were classified as high quality (see Table 1).

**Table 1:** Brughelli modified methodological quality assessment tool

Question Number	Question Criteria
Q1	Power analysis was performed and justification of study sample size given
Q2	Athlete demographics were clearly defined: gender, age, body height and body mass at the time of the test
Q3	Athlete characteristics were clearly defined: sport, experience or activity level and level of play at the time of test
Q4	Inclusion and exclusion criteria were clearly stated for athletes
Q5	Athletes or groups of athletes were similar at baseline or differences were accounted for and explained
Q6	Proper training and practice trials of the test were given to the athletes allowing for adequate familiarization
Q7	Methods were described in great detail to allow replication of the test. Testing devices, no. of trials, no. and duration of rest, speed, angle, height and test limb were included when applicable
Q8	Test–retest reliability of measurement device reported
Q9	Outcome variables were clearly defined
Q10	Statistical analyses were appropriate

### Data Extraction

Following the search process, pertinent data from included studies were extracted by two researchers. These data included: author names, publication year, country of origin (the location for the prominent affiliation of the first author), participant demographics (i.e., sample size, age, sex, years of training, athletic level [athletic level was determined according to a published participant classification framework [19] where Tier 0 is sedentary, Tier 1 is recreationally active, Tier 2 is trained/developmental, Tier 3 is highly trained/national level, Tier 4 is elite/international level, and Tier 5 is world class]), intervention details (i.e., training type, training program content, training frequency, training intensity, and intervention duration), and outcomes (i.e. variables assessed and data reported). Next, the consolidated research data were divided into five distinct training method subgroups according to previous research [21]. Specifically, training methods were classified as: reaction training (RT), which involves training activities with inherent reactions to particular stimuli (e.g., small-sided games [SSG], visual feedback training) [22]—this type of training is particularly effective in enhancing players’ cognitive abilities (open-skill); plyometric training (PT), which involves exercises that utilize the stretch-shortening cycle to enhance muscle responsiveness and power through rapid stretching and contraction (e.g., box jumps, depth jumps) [23]; strength and balance training (SBT), which involves muscle strengthening and stability training (e.g., resistance band exercises, stability ball workouts) [24]; speed training (SpT), which involves sprinting or specific linear running exercises (e.g., short sprints, interval training) [25]; and stretching training (StrT), which involves flexibility training that elongates muscles, tendons, and ligaments (e.g., static stretching, dynamic stretching) [26]. PT, SBT, SpT, and StrT are particularly effective in enhancing players’ physical abilities (closed-skill). This division enabled a comprehensive sensitivity analysis, which was designed to assess the stability and reliability of the results obtained from different training methods [27].

### Statistical Analysis

The extracted data were compiled for meta-analysis using R software (version 4.1.0). Standardized mean differences (SMD) were determined for subgroup analyses when the number of effect sizes (ES) across included studies for a specific training method was equal to or greater than three [28]. Heterogeneity was assessed using the I² statistic where *≤* 25% indicates low heterogeneity, 26–50% indicates moderate heterogeneity, 51–75% indicates high heterogeneity, and > 75% indicates substantial heterogeneity [29]. When I² values exceeded 50%, outlier values (the single ES that differs most from the combined ES) were excluded from the SMD calculation until low heterogeneity was achieved (I² <50%) [30]. In this meta-analysis, a three-level model was employed to precisely address correlations among multiple outcome measures reported within the same study, thereby overcoming the limitations of traditional two-level models and ensuring more accurate estimations of observed effects. Sensitivity analyses were conducted using a one-by-one exclusion method for each study. Cohen’s d or Hedges’ g were used to calculate the ES for changes in outcome measures between pre- and post-intervention timepoints in each study. Hedges’ g was employed when the sample size was < 20 in a study, while Cohen’s d was used when the sample size was *≥* 20 in a study. This approach was taken as a reduction in the effect > 4% has been shown using Cohen’s d compared to Hedges’ g when the sample size is < 20 [31]. The formulae adopted for SMD determined as Cohen’s d and Hedges’ g [32] were:

$$\:\text{d}=\frac{{\text{M}}_{\text{p}\text{r}\text{e}}-{\text{M}}_{\text{p}\text{o}\text{s}\text{t}}}{\sqrt{\frac{{\text{S}\text{D}}_{\text{p}\text{r}\text{e}}^{2}+{\text{S}\text{D}}_{\text{p}\text{o}\text{s}\text{t}}^{2}}{2}}}$$

$$\:\text{g}=\text{d}\:\times\:(1-\frac{3}{4\text{n}-9})$$

where M_pre_ represents the pre-intervention mean value, M_post_ denotes the post-intervention mean value, SD^2^_pre_ is the pre-intervention standard deviation, and SD^2^_post_ is the post-intervention standard deviation. ES magnitudes were classified as small (0.20–0.49), medium (0.50–0.79), or large (*≥* 0.80) [33]. To ensure consistency in calculating ES across studies adopting different tests and outcome measures with varied units, a standardized approach was employed. This approach involved reverse processing certain test scores in cases where tests were assessed by scores or the number of completions instead of completion time, effectively multiplying them by -1 [34]. When multiple ES were derived from different agility tests being administered around the same intervention, a weighted arithmetic averaging technique (combining multiple effects from the same intervention into one effect) was used to consolidate them [35]. Publication biases were assessed with funnel plots, where significant asymmetries (*p* < 0.05) indicated potential concerns about bias, necessitating a more careful interpretation of the meta-analytical findings [36]. Furthermore, Egger’s regression test was employed, where a significant intercept (*p* < 0.05) suggests the presence of publication bias. This finding casts doubt on the reliability and generalizability of the results derived from the meta-analysis [37].

## Results

### Study Selection

**Fig. 1:**
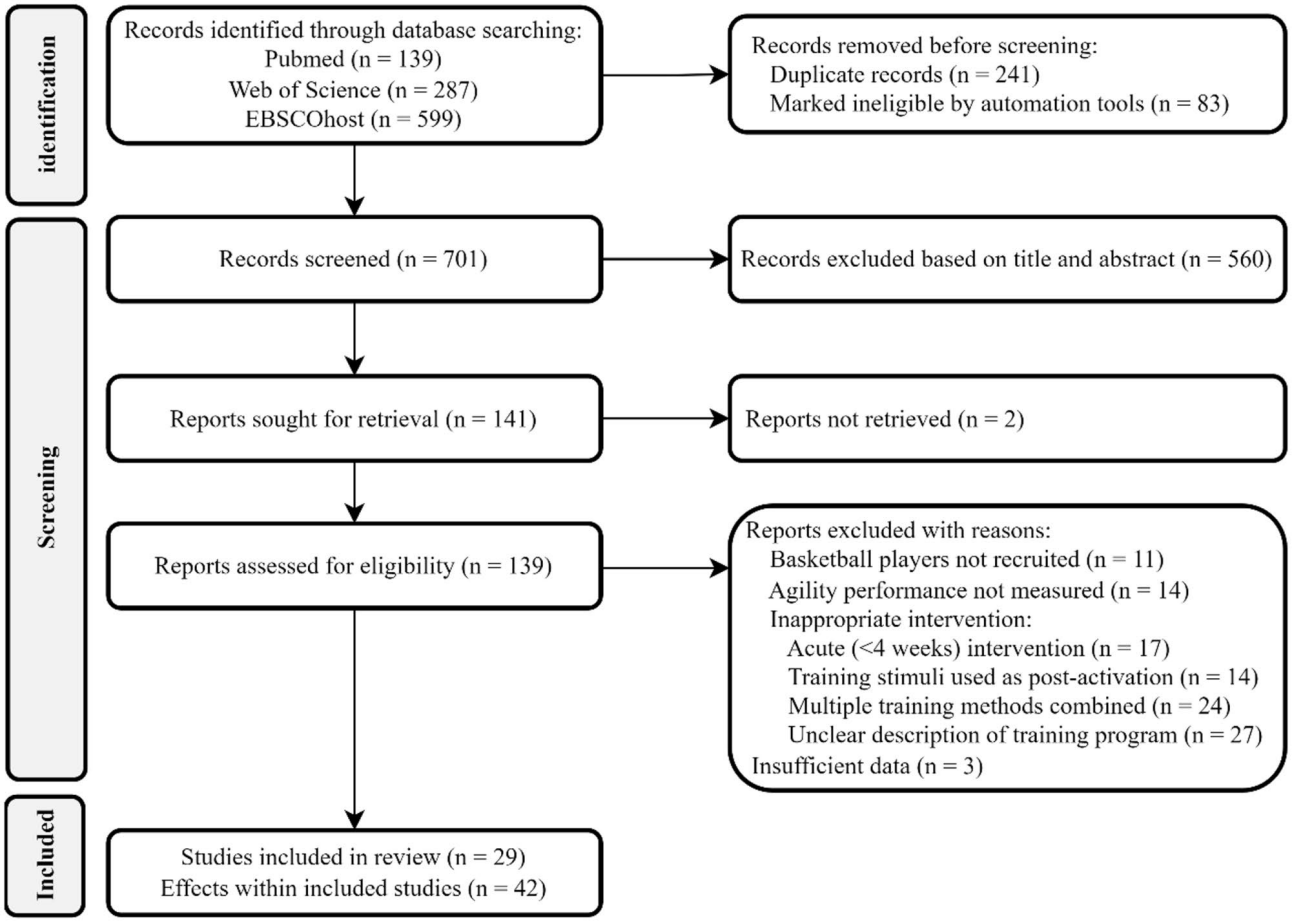
Flowchart of the literature search and selection process in this review

The search and screening process is illustrated in Fig. 1. The initial search retrieved 287 studies from Web of Science, 139 studies from PubMed, and 599 studies from EBSCOhost. After removing duplicates and applying the inclusion and exclusion criteria, a total of 29 studies were ultimately included in the review.

### Research Characteristics

The descriptive characteristics of the included studies are shown in Table 2. In turn, the quality assessment results for included studies are given in Table 3 while the descriptive summary information provided in included studies is presented in Tables 4 and 5. Participant athletic levels ranged from Tier 2 to Tier 4 (Tier 4: 20, Tier 3: 193, Tier 2: 250), with studies generated from 15 countries. Ten different types of agility tests were used to evaluate agility performance in the included studies, with the T-Test being the most common (used 18 times). All agility tests used across included studies are classified as closed-skill, with none being open-skill in nature.

**Table 2:** Descriptive characteristics of included studies included in this review

Number of studies	Number of effects	Number of participants	Intervention duration (week)	Sessions per week	Quality score
29	42	463	7.34 ± 2.82	2.72 ± 0.70	15.86 ± 1.43

**Table 3:** Quality assessment scores of included studies included in this review (see Table 1 for question criteria)

Study	Q1	Q2	Q3	Q4	Q5	Q6	Q7	Q8	Q9	Q10	Overall
Lehnert et al. [38]	0	2	1	1	1	2	2	1	2	2	14
Pinheiro et al. [39]	1	2	2	2	1	2	2	1	1	2	16
Palma-Munoz et al. [40]	2	2	2	2	2	2	2	2	1	2	19
Meszler et al. [41]	0	2	2	1	1	2	2	2	1	2	15
Cherni et al. [42]	1	2	2	1	2	1	2	2	2	2	17
Gonzalo-Skok et al. [43]	0	2	2	1	2	1	2	2	2	2	16
Ozen et al. [44]	0	2	2	1	1	1	2	2	2	2	15
Brini et al. [45]	2	2	2	2	1	2	2	2	2	2	19
Huang et al. [12]	0	2	1	2	2	0	2	2	2	2	15
Haghighi et al. [46]	2	2	2	2	1	1	2	1	2	2	17
McCormick et al. [47]	0	2	1	2	2	2	2	2	2	2	17
Maggioni et al. [48]	0	2	2	2	1	2	2	1	2	2	16
Zeng et al. [49]	0	2	2	2	2	1	2	2	2	2	17
Brini et al. [50]	0	2	2	2	1	1	2	2	2	2	16
Hassan et al. [51]	0	2	2	1	1	2	2	2	2	1	15
Arslan et al. [52]	0	2	2	1	1	2	2	2	2	2	16
Lucia et al. [53]	2	1	0	2	2	0	1	2	2	2	14
Delextrat et al. [54]	0	2	2	2	1	0	2	2	1	2	14
Arede et al. [55]	0	2	1	2	2	1	2	1	2	2	15
Atanasković et al. [56]	1	2	0	2	1	0	2	2	1	2	13
DemİRarar et al. [57]	2	2	1	2	1	2	2	2	2	2	18
Hovsepian et al. [58]	0	2	2	2	1	1	2	2	2	2	16
Bouteraa et al. [59]	0	2	2	2	1	0	2	2	2	2	15
Usgu et al. [60]	0	1	2	2	1	2	2	2	2	2	16
Chen et al. [61]	0	2	2	2	1	0	2	2	2	2	15
Okur et al. [62]	0	2	2	1	2	0	2	2	2	2	15
Arede et al. [63]	1	2	2	2	1	1	2	2	2	2	17
Suel et al. [64]	0	2	2	1	2	1	2	2	1	2	15
Cruz et al. [65]	0	2	2	2	2	1	2	2	2	2	17

**Table 4:** Descriptive summary of studies included in this review

Study	Location	Sample characteristics				Agility test	Statistical outcomes	
		Training method and sample size	Age (years)	Sex	Athletic level (Tier)		Change in test (%) with ES	*p*-value
Lehnert et al. [38]	Czech Republic	EG (PT): *N* = 11	24.36 ± 3.9	M	3	T-Test	EG = 2.14% ES = 0.40	No significant change (*p* > 0.05).
Pinheiro et al. [39]	Brazil	MEG (PT): *N* = 6 MCG: *N* = 7 FEG (PT): *N* = 11 FCG: *N* = 10	MEG: 15.83 ± 0.75 MCG: 15.43 ± 1.13 FEG: 14.45 ± 0.69 FCG: 15.30 ± 0.16	M: 16 F: 23	2	Illinois Agility Test	MEG = 3.66% ES = 0.62 MCG = 1.97% ES = 0.19 FEG = 2.29% ES = 0.49 FCG = 1.13% ES = 0.14	Significant improvements in MEG and MCG (*p* < 0.05), with no significant changes in FEG and FCG (*p* > 0.05).
Palma-Munoz et al. [40]	Chile	EG1 (PT): *N* = 7 EG2 (PT): *N* = 8 CG: *N* = 7	13.5 ± 2.0	M	2	T-Test	EG1 = 9.23% ES = 0.77 EG2 = 7.69% ES = 0.43 CG = 0 ES = 0.00	Significant improvement in EG1 and EG2 (*p* < 0.05), with no significant change in CG (*p* > 0.05).
Meszler et al. [41]	Hungary	EG (PT): *N* = 9 CG: *N* = 9	U17	F	2	Illinois Agility Test	EG=-4.57% ES=-0.69 CG = 2.91% ES=-0.52	No significant changes in EG or CG (*p* > 0.05).
						T-Test	EG=-1.55% ES=-0.27 CG = 3.11% ES=-0.47	
Cherni et al. [42]	Tunisia	EG (PT): *N* = 13 CG: *N* = 12	18–27	F	3	T-Test	EG = 3.96% ES = 0.79 CG=-1.89% ES=-0.41	Significant improvement in EG (*p* ≤ 0.001), with no significant change in CG (*p* > 0.05).
Gonzalo-Skok et al. [43]	Spain	EG1 (PT): *N* = 10 EG2 (PT): *N* = 10	U13–U14	M	4	V-cut Test	EG1 = 2.1% ES = 0.36 EG2 = 3.31% ES = 1.06	No significant improvements in EG1 and EG2 (*p* > 0.05), with EG2 was significantly greater than EG1 post-intervention (*p* < 0.05).
Ozen et al. [44]	Turkey	EG1 (PT): *N* = 6 EG2 (PT): *N* = 6	17.58 ± 0.50	M	3	Box Drill Agility Test	EG1 = 9.36% ES = 0.86 EG2 = 6.91% ES = 0.51	Significant improvements in EG1 and EG2 (*p* < 0.05); with EG1 was significantly greater than EG2 post-intervention (*p* < 0.05).
Brini et al. [45]	Iran	EG (PT): *N* = 13 CG: *N* = 13	EG1: 26.02 ± 2.37 EG2: 25.75 ± 1.76 CG: 26.35 ± 2.11	M	3	T-Test	EG1 = 0.90% ES = 0.86 EG2 = 2.09% ES = 2.14 CG = 0.30% ES = 0.41	No significant changes in EG1, EG2, or CG (*p* > 0.05).
Huang et al. [12]	China	EG (PT): *N* = 15	22.16 ± 0.85	M	2	T-Test	EG = 5.62% ES = 0.64	Significant improvement in EG (*p* < 0.05).
Haghighi et al. [46]	Iran	EG1 (PT): *N* = 8 EG2: *N* = 8 CG: *N* = 8	EG1: 14.6 ± 1.5 EG2: 15.1 ± 1.6 CG: 15.1 ± 1.8	F	3	Lane Agility Test	EG1 = 7.59% ES = 1.14 EG2 = 8.21% ES = 0.84 CG = 3.79% ES = 0.31	Significant improvements in EG1 and EG2 (*p* < 0.05), with EG1 significantly greater than EG2 post-intervention (*p* < 0.05).
McCormick et al. [47]	US	EG1 (PT): *N* = 7 EG2 (PT): *N* = 7	EG1: 16.29 ± 0.76 EG2: 15.71 ± 0.76	F	2	Right Lateral Shuffle Test	EG1 = 2.98% ES = 0.19 EG2 = 6.83% ES = 0.62	Significant improvements in EG1 and EG2 (*p* < 0.05).
						Left Lateral Shuffle Test	EG1 = 0.58% ES = 0.04 EG2 = 8.81% ES = 0.73	
Maggioni et al. [48]	Italy	EG1 (RT): *N* = 12 EG2 (SpT): *N* = 9 CG: *N* = 9	19 ± 1.0	M	3	T-Test	EG1 = 3.00% ES = 0.58 EG2 = 5.00% ES = 0.73 CG = 2.04% ES = 0.69	Significant improvements in EG1 and EG2 (*p* < 0.01).
Zeng et al. [49]	China	EG1 (RT): *N* = 9 EG2 (SpT): *N* = 10	EG1: 20.0 ± 1.3 EG2: 19.8 ± 0.8	F	2	Modified T-Test	EG1 = 6.70% ES = 1.23 EG2 = 5.43% ES = 1.13	Significant improvements in EG1 and EG2 (*p* < 0.05).
Brini et al. [50]	Tunisia	EG1 (RT): *N* = 8 EG2 (SpT): *N* = 8	23.4 ± 2.3	M	3	T-Test	EG1 = 3.33% ES = 0.33 EG2 = 4.62% ES = 0.44	Significant improvements in EG1 and EG2 (*p* < 0.001).
Hassan et al. [51]	Saudi Arabia	EG (RT): *N* = 10 CG: *N* = 10	EG:14.80 ± 0.79 CG:14.60 ± 0.70	M	2	T-Test	EG = 11.1% ES = 7.22 CG = 5.00% ES = 1.18	Significant improvements in EG and CG (*p* < 0.001).
Arslan et al. [52]	Turkey	EG1 (RT): *N* = 16 EG2 (SpT): *N* = 16	14.5 ± 0.5	M	2	T-Test	EG1 = 5.73% ES = 0.86 EG2 = 1.61% ES = 0.30	Significant improvements in EG1 and EG2 (*p* < 0.05).
Lucia et al. [53]	Italy	EG (RT): *N* = 15 CG: *N* = 15	15 ~ 17	M: 15 F: 15	2	COD Test	EG = 3.14% ES = 0.57 CG=-0.17% ES=-0.03	Significant improvement in EG (*p* < 0.05), with no significant change in CG (*p* > 0.05).
Delextrat et al. [54]	UK	EG1 (RT): *N* = 9 EG2 (SpT): *N* = 9	EG1: 16.3 ± 0.8 EG2: 16.0 ± 0.6	M	2	Control Dribble Test	EG1 = 7.19% ES = 1.39 EG2 = 4.32% ES = 0.84	Significant improvement in EG1 for both tests (*p* < 0.05). Significant improvement in EG2 for Control Dribble (*p* < 0.05), with no significant change in T-Test (*p* > 0.05).
						T-Test	EG1 = 4.54% ES = 0.66 EG2=-2.71% ES=-0.23	
Arede et al. [55]	Portugal	EG (SBT): *N* = 9 CG: *N* = 7	EG: 14.24 ± 0.35 CG: 14.89 ± 0.42	M	3	505 Test	EG = 0.72% ES = 0.20 CG=-0.70% ES=-0.11	No significant improvments in EG and CG (*p* > 0.05).
Atanasković et al. [56]	Serbia	EG (SBT): *N* = 15 CG: *N* = 15	14.56 ± 0.5	M	2	T-Test	EG = 0.11% ES = NR CG = 0% ES = NR	No significant improvements in EG and CG (*p* > 0.05).
DemİRarar et al. [57]	Turkey	EG (SBT): *N* = 13 CG: *N* = 13	12.89 ± 0.28	M	2	T-Test	EG=-1.96% ES = NR CG=-4.12% ES = NR	No significant improvements in EG and CG (*p* > 0.05).
Hovsepian et al. [58]	Iran	EG1 (SBT): *N* = 10 EG2 (SBT): *N* = 10	21.95 ± 2.45	F	3	T-Test	EG1 = 2.46% ES = 0.27 EG2 = 5.16% ES = 0.70	Significant improvements in EG1 and EG2 (*p* < 0.01).
Bouteraa et al. [59]	Tunisia	EG (SBT): *N* = 16 CG: *N* = 10	EG: 16.4 ± 0.5 CG: 16.5 ± 0.5	F	2	Modified Illinois Change of Direction Test	EG = 6.19% ES = 1.30 CG = 0% ES = 0	Significant improvement in EG (*p* < 0.001), with no significant change in CG (*p* > 0.05).
Usgu et al. [60]	Turkey	EG (SBT): *N* = 14 CG *N* = 14	EG: 26.6 ± 5.9 CG:22.4 ± 4.2	M	3	T-Test	EG = 3.78% ES = 0.84 CG = 0.95% ES = 0.19	Significant improvement in EG (*p* < 0.01), with no significant change in CG (*p* > 0.05).
Chen et al. [61]	China	EG1 (SBT): *N* = 15 EG2 (SpT): *N* = 15	EG1: 21.1 ± 1.7 EG2: 20.6 ± 1.8	M	3	T-Test	EG1 = 0% ES = 0 EG2=-2.06% ES=-0.31	No significant improvements in EG1 or EG2 (*p* > 0.05).
Okur et al. [62]	Turkey	EG (SpT): *N* = 13 CG: *N* = 13	15.35 ± 0.49	M	2	T-Test	EG = 1.25% ES = 0.32 CG = 0.93% ES = 0.35	Significant improvements in EG and CG (*p* < 0.05), with no significant inter-group differences (*p* > 0.05).
Arede et al. [63]	Portugal	EG1 (SpT): *N* = 7 EG2 (SpT): *N* = 6 EG3 (SpT): *N* = 16	EG1: 12.01 ± 0.36 EG2: 13.32 ± 0.58 EG3: 16.97 ± 1.15	M	2	Modified 505 Test	EG1 = 1.91% ES = 0.13 EG2 = 3.22% ES = 0.45 EG3 = 2.15% ES = 0.20	No significant improvements in EG1, EG2, and EG3 (*p* > 0.05).
Suel et al. [64]	Turkey	EG (SpT): *N* = 10	20.6 ± 1.6	F	3	T-Test	EG = 7.65% ES = 1.46	Significant improvement in EG (*p* < 0.05).
Cruz et al. [65]	USA	EG (SpT): *N* = 8 CG: *N* = 7	15.7 ± 0.8	M	3	Shuttle Run Test	EG = 0% ES = 0 CG=-1.02 ES=-0.27	No significant improvements in EG or CG (*p* > 0.05).

*Abbreviations*: M, male; F, female; U, under; EG, experimental group; EG1, experimental group 1; EG2, experimental group 2; CG, control group; MEG, male experimental group; FEG, female experimental group; FCG, female control group; ES, effect size; PT: plyometric training; RT: reaction training; SBT: strength and balance training; SpT: speed training

**Table 5:** Intervention design of studies included in this review

Study	Duration (weeks)	Frequency (sessions/week)	Design
Lehnert et al. [38]	6	2 sessions for weeks 1–4, 4 sessions for week 5–6	The training included: Part A for weeks 1, 2, and 3: 1–3 sets × 3–12 repetitions of squat jumps, single-leg hurdle jumps, and medicine ball chest passes. Part B for weeks 1, 3, and 4: 2–3 sets × 4–6 repetitions of abdominal jumps, split jumps, and medicine ball rotational relays. Part C for weeks 2 and 3: 2–4 sets × 4–10 repetitions of scissor jumps, bilateral forward jumps, and medicine ball rotational throws. Throughout all parts, 2 min of rest was given between each set.
Pinheiro et al. [39]	6	3	MEG and FEG included vertical, lateral, and standing long jumps, abdominal jumps, split squat jumps, continuous single-leg forward jumps, and single-leg vertical and lateral jumps. Sessions consisted of 50 total repetitions in the first week and increased by 10 repetitions weekly. Rest periods were 30 s between jumps and 2 min between sets. MCG and FCG followed regular basketball training routines.
Palma-Munoz et al. [40]	6	2	EG1 and EG2 included 2 sets × 2 series × 5 repetitions of single-leg and double-leg forward jumps as well as vertical jumps. In EG2, the number of repetitions per set increased by 1 every 2 weeks. The CG followed regular basketball training routines.
Meszler et al. [41]	7	2	EG included 2–6 sets × 4–10 repetitions of single-leg and double-leg hurdle jumps, single-leg and double-leg lateral cone jumps, single-leg forward jumps, and double-leg depth jumps. CG followed regular basketball-specific training.
Cherni et al. [42]	8	2	EG included 4 sets × 6 repetitions of stride jumps, hurdle jumps, and depth jumps, with the number of sets increasing by 1 every 2 weeks. CG followed regular basketball training routines.
Gonzalo-Skok et al. [43]	6	2	EG1 included bilateral vertical training, encompassing 2–3 sets × 5 repetitions of 20-cm drop jumps, arm swing squat jumps, arm swing squat hops, group jumps, and hurdle jumps. EG2 engaged in unilateral horizontal training, consisting of 2–3 sets × 5 repetitions of 10-cm depth jumps, standing long jumps, no squat standing long jumps, triple jumps, and 5 sets × 2–4 repetitions unilateral jumps. Excluding unilateral jumps, the volume for other exercises increased by 1 set every 2 weeks.
Ozen et al. [44]	6	3	Both EG1 and EG2 included 3 sets × 8 repetitions of vertical jumps, standing long jumps, single-leg squat jumps, repetitive squat jumps, broad jumps, and depth jumps, with the repetitions per set increasing by 2 each 2 weeks. EG1 practiced on a sand surface (20-cm depth), while EG2 conducted exercises on a standard wooden floor.
Brini et al. [45]	8	2	EG1 included 3 sets × 10 repetitions of 50-cm drop jumps for the first month and 3 sets × 12 repetitions for the second month. Recovery times between repetitions were 40 s and between sets were 3 min. EG2 consisted of 3 sets × 8 repetitions of 30-m (6 × 5 m) sprints for the first month and 3 sets × 10 repetitions for the second month. Rest periods were 30 s between repetitions and 4 min between sets. CG followed regular basketball training routines.
Huang et al. [12]	8	3	EG incorporating vertical jumps, horizontal jumps, and full-body weight exercises with intensities ranging from 4.0 to 6.0 metabolic equivalents, and repetitions from 10 to 6 per set with increasing intensity.
Haghighi et al. [46]	6	3	EG1 included PT jumps training with 105–174 repetitions per session. EG2 engaged in repeated high-intensity interval strength and sprint training lasting 5–10 min per session. CG maintained regular basketball training.
McCormick et al. [47]	6	2	EG1 included sagittal PT ankle hops and squat jumps (weeks 1–3), and continuous hops and split squat jumps (weeks 4–6). EG2 focused on frontal contractions with exercises like lateral hops and skating drills (weeks 1–3), and continuous lateral hops and bounds (weeks 4–6). Rest periods were 60 s between sets and 15 s between exercises during weeks 1–3 and 60–90 s between sets and 30 s between exercises during weeks 4–6, with all exercises performed in 4 sets × 6 repetitions.
Maggioni et al. [48]	8	3	EG1 included 3 sets × 4-min half-court 3vs3 matches, with 1-min breaks, totaling 6 ± 2 h per week. EG2 executed 3 sets × 6 repetitions × 40-m (20 m + 20 m) sprints, with rest periods of 20 s between repetitions and 3 min between sets. CG followed regular basketball training routines.
Zeng et al. [49]	4	3	EG1 engaged in 2vs2 half-court matches in week 1 at 3 sets × 2 periods × 2 min 45 s, weeks 2–3 at 3 sets × 2 periods × 3 min 15–45 s), week 4 at 3 sets of 3 periods × 2 min 40 s with 2 min of rest between sets. EG2 performed 3 sets of 6 min of sprints at 90% of speed attained in final stage of 30 − 15 Intermittent Fitness Test, increasing by 1 min per set biweekly, and speed escalating to 95% during weeks 3–4.
Brini et al. [50]	4	2	EG1 included competitive high-intensity 2vs2 training on a half-sized field (28 m × 7.5 m) in weeks 1–2 at 2 sets × 2 periods × 3 min 45 s and weeks 3–4 at 2 sets × 3 × 4 min 30 s. EG2 included 3 sets of 6 maximal 30-m (15 m + 15 m) sprints for weeks 1–2, increasing to 8 repetitions in weeks 3–4, with 20-second inter-repetition and 4-min interset rest.
Hassan et al. [51]	8	4	EG included FITLIGHT reactive agility with or without ball training for 2–3 sets × 2–8 repetitions, with 2–4 min of rest between sets. CG followed regular basketball training routines.
Arslan et al. [52]	6	3	EG1 consisted 2 × 2 repetitions × 2 minutes 30 seconds to 4 minutes of full-court 2vs2 SSG, with 2 minutes of passive rest between each bout. EG2 included 2 × 6–9 minutes of 15”–15”at 90–95% of velocity at intermittent fitness test (VIFT), with 15 s of rest between repetitions.
Lucia et al. [53]	5	3	EG included reactive training based on visual feedback. CG followed regular physical fitness and sprint training.
Delextrat et al. [54]	6	2	EG1 included high-intensity 2vs2 training on a half-sized field (28 m × 7.5 m), consisting of 2 sets × 2–3 periods × 3 min to 4 min 15 s. EG2 included 2 sets × 8–13 min of sprints at 95% of speed attained in final stage of 30 − 15 Intermittent Fitness Test, with an active recovery of 15 s between sets.
Arede et al. [55]	8	4	EG included stability, neuromuscular, and explosive training. The neuromuscular circuit training consisted of three stations, conducted in 2 sets × 90 s, with no rest between repetitions or sets. The explosive training component included three types of resistance training, followed by multi-dimensional band resistance training using the VertiMax V6. The CG followed regular basketball training routines.
Atanasković et al. [56]	6	3	EG included vibration training at 3 sets × 8 repetitions × 40 s where frequencies were 20 Hz, 25 Hz, and 30 Hz for the first, second, and third sets respectively, with 1 mm amplitude, with rest periods of 1 min between repetitions and 2 min between sets. Amplitude increased by 1 mm weekly. CG followed regular basketball training routines.
DemİRarar et al. [57]	8	3	EG included suspension control training, maintaining a 60° body-to-ground angle for weeks 1–4, performing 1 set × 30 s of seated chest presses, standing tricep overhead extensions, dumbbell curls, supine suspended arm flexions, planks, squats, and suspended leg curls. The angle was adjusted to 50° for weeks 5–8. CG followed regular basketball training routines.
Hovsepian et al. [58]	10	4	EG1 included strength and coordination training, incorporating 75–85% of 1RM weight in isolated or combined movements for strength, with speed, agility, and defensive footwork exercises for coordination. EG2 engaged in strength, endurance, and metabolic conditioning, working at 70–90% of maximum heart rate and 60–70% of 1RM per repetition.
Bouteraa et al. [59]	8	2	EG included 2–3 sets × 11–15 supramaximal contractions and 6–10 sets × 20–30 balance exercises, including vertical, forward, depth (40–60 cm), hurdle (40–60 cm), and “Z” jumps, with 90 s of rest between repetitions. Balance drills included kneeling Swiss ball, chest pass, and single-leg exercises, with 30-second inter-repetition rest. CG followed regular basketball training.
Usgu et al. [60]	20	2	EG included five stages of functional training, including bench press, sit-ups, double leg lifts, hip bridge, and Russian twists. CG engaged in varied position strength training, increasing upper limb load and overall weight by 5% and 10% monthly, respectively.
Chen et al. [61]	8	3	EG1 included battle rope training with work-to-rest ratios of 1:3 for weeks 1–2 and 1:2 for weeks 3–8. EG2 performed 15-m 180° shuttle sprints at 75–85% of maximal speed, with work-to-rest ratios of 1:3 for weeks 1–2 and 1:2 for weeks 3–8.
Okur et al. [62]	8	3	EG included 3–6 sets × 10–60-m accelerations and sprints at 80–90% of maximal speed, and 4–6 sets of jumping and balance training. CG followed regular basketball training routines.
[63]	9	2	EG1, EG2 and EG3 all included 2 sets × 10 repetitions × 20-m sprints, with rest periods of 30 s between repetitions and 3 min between sets.
Suel et al. [64]	8	4	EG included ball-integrated drills at 4 stations in 2 series of 3 sets × 5 repetitions. Station 1 included dribbling followed by a sprint. Station 2 included directional changes and rebounding within a timed sequence. Station 3 included dribbling, jumps, and shots. Station 4 continued to develop shooting skills after change-of-direction dribbles. Rest periods of 30 s between repetitions, 3 min between sets, and 8 min between series rest were applied.
Cruz et al. [65]	6	2	EG included 7 Pilates exercises in 2–3 sets × 15–20 repetitions, with 45-second rest periods between sets. CG followed regular basketball training routines.

*Abbreviations*: EG, experimental group; EG1, experimental group 1; EG2, experimental group 2; CG, control group; SSG, small-sided games; MEG, male experimental group; FEG, female experimental group; RM, repetition maximum

### Main Findings

**Fig. 2:**
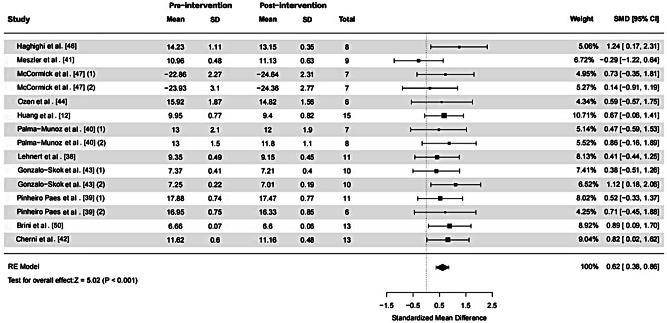
The effect of plyometric training on closed-skill agility performance in basketball players. *Abbreviations*: SD, standard deviation; SMD, standardized mean difference; 95% CI, 95% confidence intervals; RE, random effects. *Note*: (1) and (2) represent different effect sizes (ES) derived from the same study, where multiple interventions or subgroups were analyzed separately

**Fig. 3:**
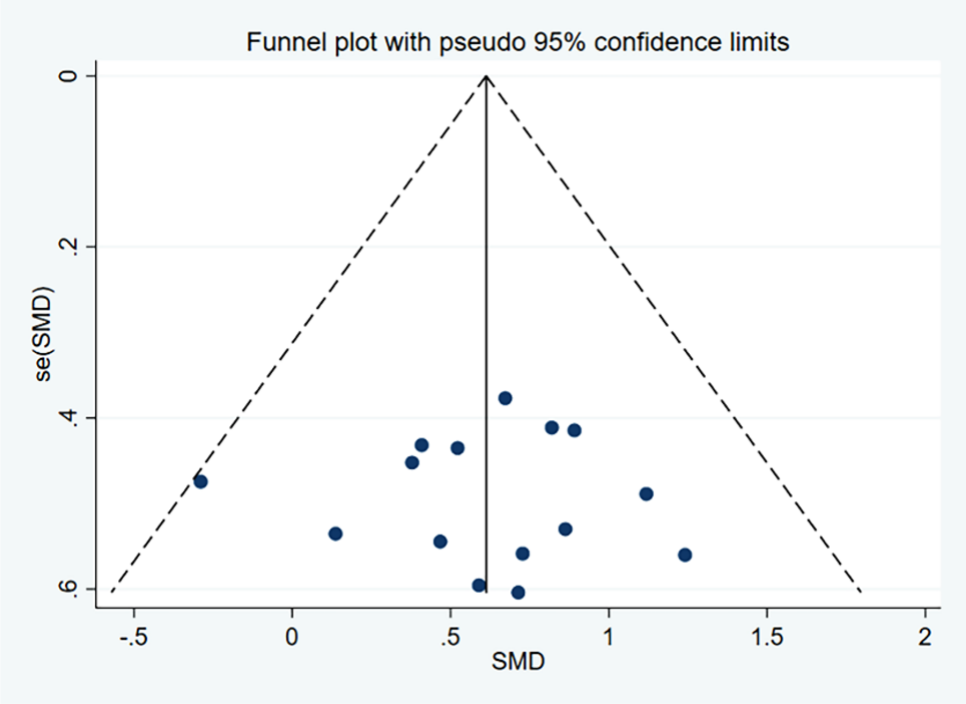
Funnel plot of the publication bias in studies examining the effects of plyometric training on closed-skill agility performance in basketball players. *Abbreviations*: SMD, Standardized Mean Difference; SE(SMD), Standard Error of the Standardized Mean Difference

15 ES and 141 samples were included in the calculation of the SMD for PT, as illustrated in Fig. 2. Low heterogeneity was detected (χ^2^ = 8.49; *p* = 0.862, I^2^ = 0%). The funnel plot analysis for publication bias, as presented in Fig. 3, demonstrated near symmetry, while Egger’s test indicated no significant publication bias (*p* = 0.913), suggesting relatively credible results. The calculated SMD was 0.62 (95%CI = 0.38, 0.86), indicating a medium ES. The primary drill utilized across studies in administering PT sessions was vertical jump training (10 out of 11 studies).

**Fig. 4:**
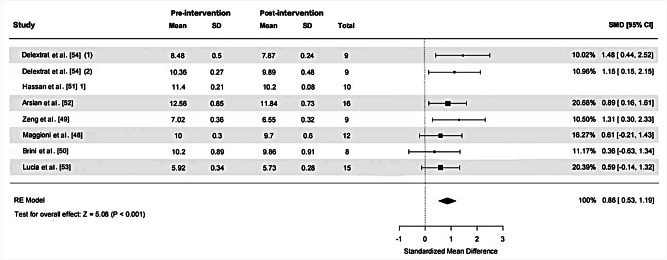
The effect of reaction training on closed-skill agility performance in basketball players. *Abbreviations*: SD, standard deviation; SMD, standardized mean difference; 95% CI, 95% confidence intervals; RE, random effects. *Note*: (1) and (2) represent different effect sizes (ES) derived from the same study, where multiple interventions or subgroups were analyzed separately

**Fig. 5:**
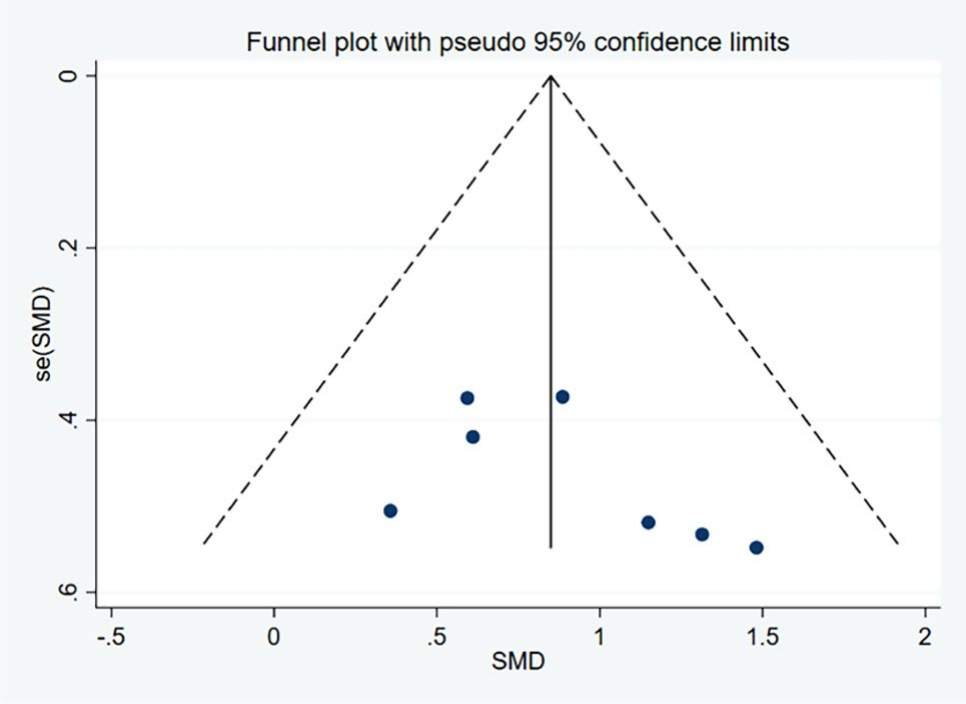
Funnel plot of the publication bias in studies examining the effects of reaction training on closed-skill agility performance in basketball players. *Abbreviations*: SMD, Standardized Mean Difference; SE(SMD), Standard Error of the Standardized Mean Difference

Eight ES and 88 samples were included in the calculation of the SMD for RT, as depicted in Fig. 4. High heterogeneity was detected (χ^2^ = 31.61, *p* < 0.001, I ^2^=94.58%). A sensitivity analysis was conducted by excluding an outlier (ES = 7.65), following which heterogeneity was rated as low (χ^2^ = 4.32, *p* = 0.634, I ^2^=0%). The funnel plot analysis for publication bias, illustrated in Fig. 5, was approaching symmetry, with Egger’s test indicating no significant publication bias (*p* = 0.198), indicating relatively credible results. The calculated SMD was 0.86 (95%CI = 0.53, 1.19) demonstrating a large ES. The primary drill utilized across studies administering RT sessions was SSG (5 out of 7 studies), particularly with 2vs2 player configurations [49, 50, 54].

**Fig. 6:**
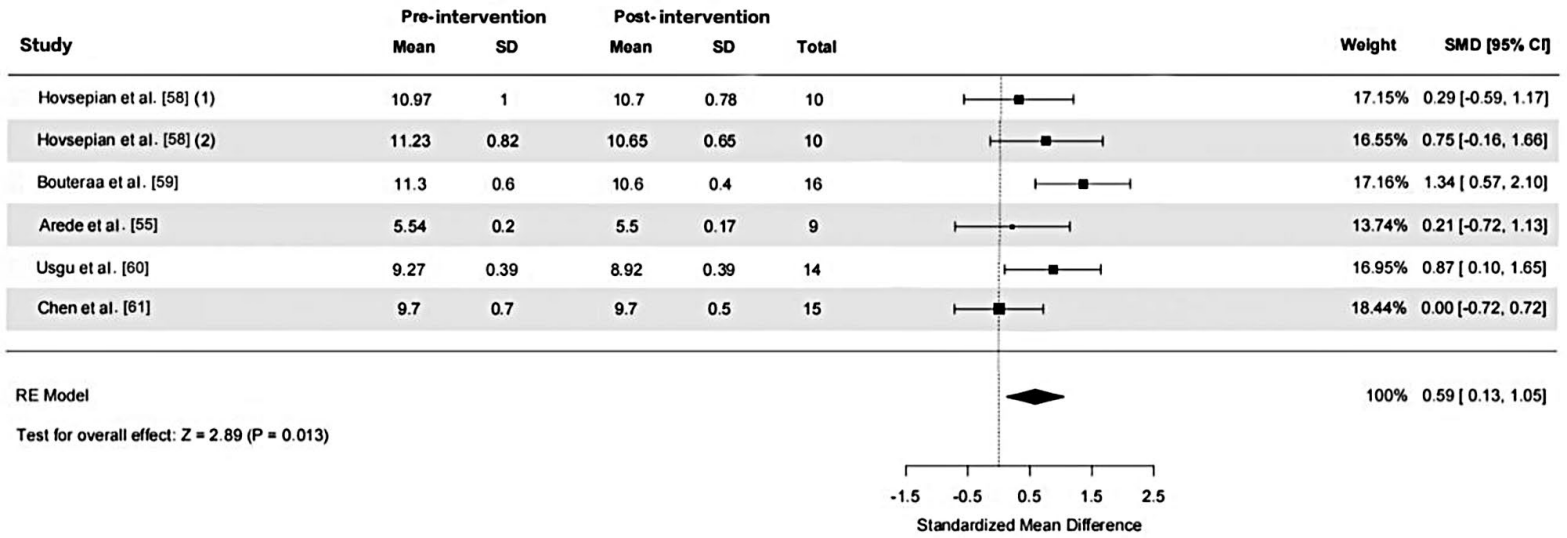
The effect of strength and balance training on closed-skill agility performance in basketball players. *Abbreviations*: SD, standard deviation; SMD, standardized mean difference; 95% CI, 95% confidence intervals; RE, random effects. *Note*: (1) and (2) represent different effect sizes (ES) derived from the same study, where multiple interventions or subgroups were analyzed separately

**Fig. 7:**
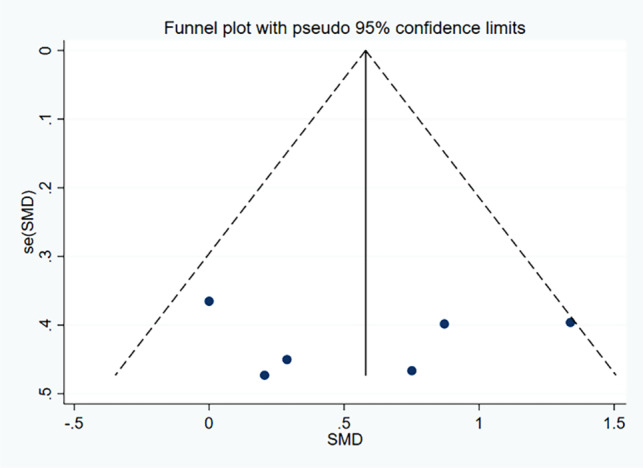
Funnel plot of the publication bias in studies examining the effects of strength and balance training on closed-skill agility performance in basketball players. *Abbreviations*: SMD, Standardized Mean Difference; SE(SMD), Standard Error of the Standardized Mean Difference

Six ES and 74 samples were included in the calculation of the SMD for SBT, as illustrated in Fig. 6. Moderate heterogeneity was detected (χ^2^ = 8.01, *P* = 0.156, I ^2^=42.6%). The funnel plot analysis for publication bias, depicted in Fig. 7, demonstrated near symmetry, while Egger’s test indicated no significant publication bias (*p* = 0.972), suggesting relatively credible results. The calculated SMD was 0.59 (95%CI = 0.13, 1.05) indicating a medium ES. The primary drill utilized across studies administering SBT sessions was functional training (4 out of 5 studies).

**Fig. 8:**
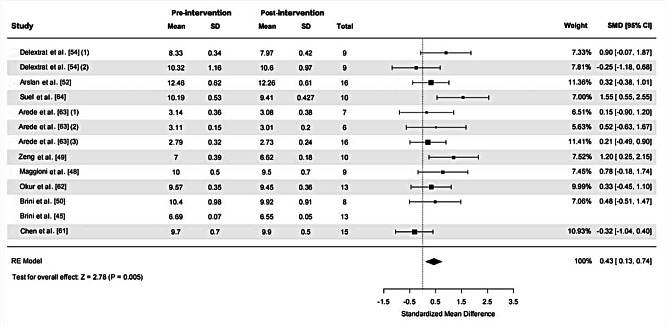
The effect of speed training on closed-skill agility performance in basketball players. *Abbreviations*: SD, standard deviation; SMD, standardized mean difference; 95% CI, 95% confidence intervals; RE, random effects. *Note*: (1), (2) and (3) represent different effect sizes (ES) derived from the same study, where multiple interventions or subgroups were analyzed separately

**Fig. 9:**
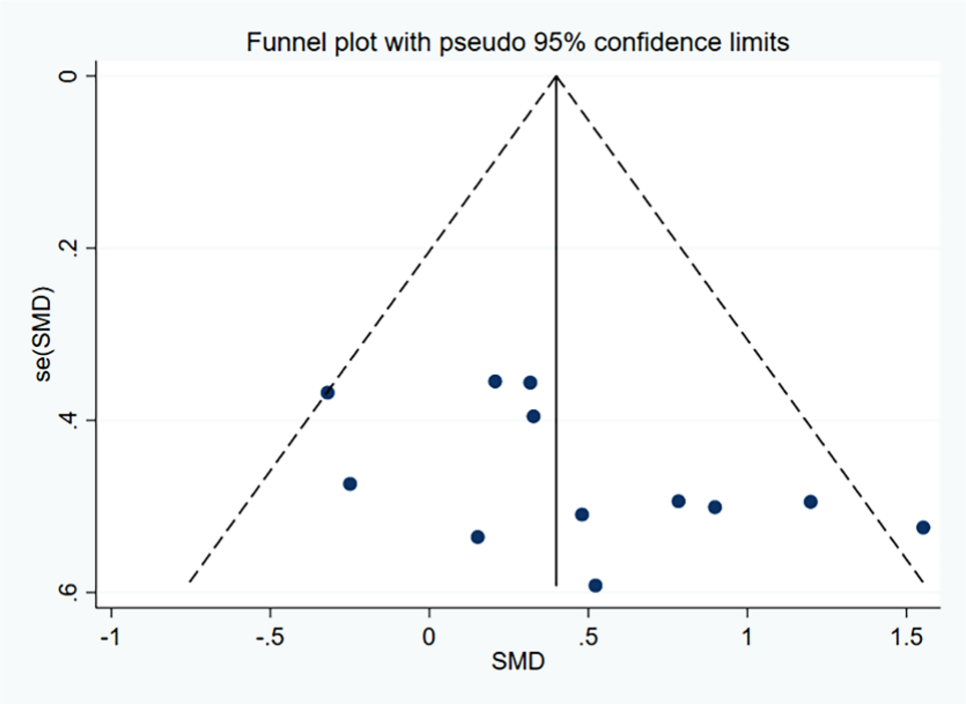
Funnel plot of the publication bias in studies examining the effects of speed training on closed-skill agility performance in basketball players. *Abbreviations*: SMD, Standardized Mean Difference; SE(SMD), Standard Error of the Standardized Mean Difference Only one study [65] was included in the StrT analysis, where the SMD was 0 (with 95%CI of -0.98, 0.98). The results indicated no significant improvement in agility performance in the EG (*p* > 0.05), and no significant difference was observed between the EG and CG groups

Thirteen ES and 141 samples were included in the calculation of SMD for SpT, as depicted in Fig. 8. High heterogeneity was detected (χ^2^ = 28.31, *P* = 0.005, I ^2^=61.5%). Sensitivity analysis was conducted by excluding an outlier (ES = 2.23), after which heterogeneity was rated as moderate (χ^2^ = 15.81, *P* = 0.148, I ^2^=30.6%). The funnel plot analysis for publication bias, represented in Fig. 9, approached symmetry, with Egger’s test indicating no significant publication bias (*p* = 0.079), suggesting relatively trustworthy results. The calculated SMD was 0.43 (95%CI = 0.13, 0.74) demonstrating a small ES. The primary drill utilized across studies examining SpT sessions was high-intensity interval training (7 out of 10 studies).

## Discussion

Identifying the most effective training methods is highly sought by basketball coaches and performance staff to optimize specific physical fitness attributes in their players [66]. In basketball, previous studies on agility training have predominantly focused on physical and coordinative aspects, often overlooking the cognitive dimension. The inclusion of both open-skill and closed-skill aspects in this review represents a novel contribution to the field. Accordingly, this review aimed to provide a comprehensive synthesis of studies conducted in the past decade to quantify the precise impact of different training methods on open-skill and closed-skill agility performance among basketball players. Importantly, the findings revealed that RT was the most effective method in enhancing closed-skill agility performance in basketball players. In turn, PT and SBT were both relatively effective in improving closed-skill agility performance, with SBT being slightly better. Finally, SpT had relatively limited effects on closed-skill agility performance, with insufficient evidence available to draw conclusions on the effects of StrT. Surprisingly, all included studies assessed agility performance using closed-skill tests (eight tests in total with the T-Test being most commonly adopted), with none using open-skill agility tests.

The superior effects of RT on closed-skill agility performance that we observed (SMD = 0.86, large ES, 88 participants) were based on seven studies examining eight ES. Notably, RT had the shortest average intervention duration of 5.88 ± 1.55 weeks across studies among the training methods examined, which shows that RT appears to be relatively efficient alongside being effective for developing agility performance in basketball players. The benefits of RT on closed-skill agility performance in basketball might be attributed to enhanced neuromuscular adaptation and better muscle recruitment patterns [67]. With improved neuromuscular efficiency, RT can enhance the speed and responsiveness of muscle contractions during basketball-specific movements [68]. Some RT drills such as SSG, which was the most predominant form of RT across studies, likely involved multi-directional movement patterns where players were required to cut and change direction to create space from opponents on offense and use lateral shuffles and runs to follow opponents on defense. These movements may directly correspond to the types of tests employed, such as the T-Test, which includes similar movements. Moreover, refined muscle recruitment strategies underpinning RT, with a focus on training fast-twitch muscle fibers, enable rapid activation of various muscle groups to promote faster, more precise, and explosive execution of specific movements [69]. In line with our findings, a previous systematic review exploring the effects of various training methods on agility performance reported that RT methods led to the greatest improvements in agility performance (7.2–19.0%) across 5 studies examining 150 basketball players collectively [13]. Consequently, these findings combined with our results emphasize that RT should be prioritized to enhance closed-skill agility performance in basketball players.

PT is a popularized training method in basketball that integrates biomechanical and neurophysiological principles for enhancing physical performance and reducing injury risk [70, 71]. PT involves rapid muscle contractions through activation of fast-twitch muscle fibers to ultimately improve force production through enhanced muscle elasticity and stretch reflex [72]. Additionally, PT promotes neural adaptations like improved motor-unit recruitment and neuromuscular coordination [72, 73], as well as optimizes the phosphagen system for immediate energy provision during explosive movements [74, 75]. These collective benefits of PT likely underpin the improvements we observed in closed-skill agility performance (SMD = 0.62, medium ES, 141 participants) among basketball players, based on 11 studies and 15 effects. Indeed, the primary drill utilized across studies examining PT in our review was vertical jump training, while the primary test utilized to assess closed-skill agility in these studies was the T-Test. Accordingly, various forms of vertical jump tasks, such as the squat jump (R^2^ = 59%) [76], countermovement jump (*r* = -0.69) [77], drop jump (*r* = -0.54) [77], and horizontal jump (R^2^ = 45%) [78], have been shown to significantly correlate with T-Test performance in basketball players, suggesting that mechanical and functional aspects of jump performances strongly translate to basketball-specific closed-skill agility maneuvers. Indeed, a previous systematic review exploring the effects of various training methods on agility performance (without quantitative analyses or discerning between open-skill and closed-skill agility) documented improvements ranging from 2.34 to 6.79% with PT across 12 studies examining 195 basketball players in total [13]. Similarly, a systematic review with meta-analysis focused solely on the effects of PT on various physical fitness attributes showed that PT significantly improved closed-skill agility performance in tests *≤* 40 m in distance (*p* < 0.001, ES (Hedges’ g) = 1.15 across 15 effects) and > 40 m in distance (*p* = 0.006, ES = 1.02 across 6 effects) among basketball players [79]. Despite the relatively short average intervention duration of 6.47 ± 0.83 weeks across studies examining PT in our review, it has previously been shown that there are no further significant improvements in closed-skill agility with longer interventions when analysed among those lasting < 8 weeks and those lasting *≥* 8 weeks in basketball players [79]. We also had varied samples of players grouped together when determining the SMD across studies examining the effects of PT on agility, with the low heterogeneity observed suggesting the effects were relatively robust in light of variations in player age, sex, and athletic level. Supporting this notion, consistent effects of PT on closed-skill agility have been observed among players irrespective of variations in key anthropometric attributes (i.e., males vs. females; stature of *≤* 174 cm vs. >174 cm; and body mass of *≤* 65.8 kg vs. >65.8 kg) [79].

In basketball competitions, players are required to consistently maintain dynamic control and balance of body posture during intense movements encompassing multidirectional accelerations, decelerations, and directional changes [56]. This emphasis on dynamic control underlines the importance of players possessing adequate strength and balance attributes to execute change-of-direction maneuvers. More precisely, where players can apply greater forces to the ground in the opposite direction to the current motion, more effective acceleration can be achieved following a change-in-direction [80]. In turn, training that enhances eccentric contraction force production in players reduces deceleration time during directional changes, allowing for a faster transition to the push-off phase and increased concentric contraction force [81]. Consequently, these mechanisms may explain the medium ES (SMD = 0.59, 74 participants) we observed for SBT in improving closed-skill agility performance in basketball players. Certainly, the varied functional styles of training adopted across studies implementing SBT included in our review encompassed various movements involving multifaceted contraction types across several muscle groups to promote wide-ranging strength and balance development. In support of the varied functional training drills adopted across studies, many strength properties, including adductor and abductor isometric strength [77], lower-body eccentric strength [1], and whole-body functional strength [76], as well as dynamic balance [82] and stability [83] indices have been shown to significantly contribute to T-Test performance in basketball players.

Unlike the other training methods examined in our review, SpT yielded only a small improvement in closed-skill agility performance (SMD = 0.43, 141 participants) based on 10 studies and 13 effects. This finding suggests that solely incorporating speed-related drills produces limited benefit to closed-skill agility in basketball players. Traditionally, speed is defined as the ability to move across a fixed distance in the shortest possible time, excluding any specific directional shifts [84]. However, basketball activity rarely requires players to move at high speeds without changing direction [82]. In support of this notion, Ziv and Lidor highlighted that only 5% of sprints last longer than 4 s during basketball games with players seldom reaching their peak speed, thereby emphasizing the strong requirement to decelerate, change direction, and re-accelerate during basketball game-play [85]. In turn, the absence of deceleration components and lateral movements in sprint-based HIIT drills, as predominantly used across studies examining SpT in our review, may limit the transference of performance-related adaptations to closed-skill agility movements [80]. Considering these findings, we suggest training regimes match the distinctive movement dynamics of basketball when performed at speed, to maximize the efficacy of adaptations to closed-skill agility performance given that SpT appears to exert limited impact.

StrT is a common drill in specialized basketball training, typically conducted both before and after training sessions, to improve athletic performance, reduce injury risk, and facilitate functional recovery [86, 87]. Although long-term StrT has the potential to influence the agility performance of basketball players [60], only one study examining StrT was included in our review. The intervention consisted of a 6-week Pilates training program, with an ES of 0 (95% CI of -0.98, 0.98) [65]. It might be stated that StrT seems to have a minimal impact on the agility performance of basketball players, although much more comprehensive research is needed to expand upon these initial findings to confirm them.

### Limitations and Recommendations for Future Research

In the experimental studies included in this systematic review and meta-analysis, variations in the athletic level, sex, and age of players examined may have influenced the meta-analysis results for different training methods. Additionally, differences in the duration of training interventions might also impact the outcomes. Therefore, as further evidence emerges, it is imperative to synthesize findings regarding the effects of different training methods on agility performance according to key player and training factors for greater specificity in outcomes. Given the differences in cognitive and physical adaptability across various basketball skill levels, the findings of this study suggest that training interventions should be tailored accordingly. Young athletes may benefit more from drills emphasizing reactive agility and decision-making, while elite players might require more complex, game-specific scenarios to further enhance cognitive agility. Furthermore, research published in the past decade examining the effects of training interventions on agility performance exclusively used closed-skill agility tests, neglecting the impact of different training methods on open-skill agility. Therefore, future studies are needed exploring how different training methods influence open-skill agility performance in basketball players using suitable tests that incorporate cognitive and physical elements.

## Conclusion

In synthesizing research findings on the effects of various training methods on agility performance in basketball from the past decade, we found that specialized training approaches are critical for optimal closed-skill agility performance in basketball. In this regard, RT predominantly in the form of SSG drills, offered the greatest benefit (large ES) to closed-skill agility performance in basketball players. In turn, PT, predominantly in the form of jump training, and SBT, predominantly in the form of functional training, were also effective (medium ES) in enhancing closed-skill agility performance in basketball players. SpT training, predominantly in the form of HIIT drills, offered limited benefit (small ES) to closed-skill agility performance in basketball players, with insufficient evidence available for StrT to draw definitive conclusions. Based on these findings, it is recommended that basketball coaches and performance staff emphasize RT content and integrate various PT and SBT methods as suited to the team context to optimize closed-skill agility performance in their players. A surprising finding from our review was that no included studies assessed the effects of training interventions on open-skill agility performance, showing a need for future research in this area.

## Data Availability

All data will be made available on request to the corresponding author.

## Abbreviations

RT: Reaction training
SpT: Speed training
SBT: Strength and balance training
PT: Plyometric training
StrT: Stretching training
COD: Change-of-direction
SD: Standard deviation
SMD: Standardized mean differences
CI: Confidence interval
RE: Random effects
ES: Effect size
SSG: Small-sided games
HIIT: High intensity interval training
RM: Repetition maximum
